# Primary school teachers’ contributions to oral health promotion in urban and rural areas of the Gulu District, Northern Uganda: a qualitative study

**DOI:** 10.1186/s12903-022-02239-6

**Published:** 2022-05-28

**Authors:** Peter Akera, Sean E. Kennedy, Mark J. Obwolo, Aletta E. Schutte, Raghu Lingam, Robyn Richmond

**Affiliations:** 1 School of Population Health, University of New South Wales, Sydney, Australia; 2 School of Women’s and Children’s Health, University of New South Wales, Sydney, Australia; 3 Department of Public Health, Faculty of Medicine, Gulu University, P.O. Box 166, Gulu City, Uganda; 4 The George Institute for Global Health, Sydney, Australia

**Keywords:** Dental caries, Primary school, School teacher, Oral health, Health promoting school framework

## Abstract

**Background:**

Dental caries remains the most prevalent non-communicable disease globally affecting 60–90% of children. The World Health Organisation’s (WHO) health-promoting school program offers a framework for dental intervention in low- and middle-income countries (LMICs). This study explored teacher contributions to children’s oral health in relation to the WHO health-promoting school framework in rural Uganda.

**Methods:**

Semi structured interviews were conducted with a purposive sample of 18 teachers. All interviews were transcribed verbatim and analysed thematically.

**Results:**

Many teachers reported preparing children to practise proper oral hygiene care through skills training and demonstrations around proper teeth brushing. Teachers’ roles included raising health awareness by providing information on oral health topics using different educational methods. Many teachers mentioned performing oral health examinations on children at the school, first aid, referral for dental treatments and engaging parents, students and health workers in oral health promotion.

**Conclusions:**

Teachers play an essential role in oral health promotion in countries like Uganda. Teachers are implementing key principles of the WHO’s health-promoting school framework on the ground and need to be considered as a key public health resource. If improvements in oral health are to be attained in Sub-Saharan Africa and other LMICs, government interventions need to harness teachers’ contributions in delivering oral health promotion.

## Background

Dental caries remains the most prevalent non-communicable diseases (NCD) globally, affecting 60–90% of children [1]. Children suffer from tooth infection, pain, tooth loss, nutritional problems and missed days from school due to poor oral health [2, 3]. Though we have known about the morbidity associated with poor oral health, over the last 30 years there have been no improvements in oral health globally. Between 1990 and 2017 the global prevalence of caries in permanent teeth in all age groups increased by 35.9% (95% UI, 33.5–38.3%), with lower income countries having a greater increase in number of prevalent cases [4]. In low- and middle-income countries (LMICs), like Uganda, the prevalence of dental caries is concerning and is expected to increase due to the increase in the consumption of unhealthy diets high in sugar and inadequate exposure to fluorides [3].

In 1995, the World Health Organisation (WHO) introduced the approach of promoting the general health of students, staff, parents and the community through schools. In 2007, this was adopted nationally and locally in Gulu, with implementation of a school oral health program that includes oral health education, periodic screening and training teachers on oral health education and promotion [5].

Teachers contribute to the design and implementation of oral health programs in schools. Teachers are health promoters, educators, instructors, inspectors and role models [6, 7]. The WHO recommends that teachers should participate in the planning, development and review of oral health policy; training in oral health and prevention of oral diseases; monitoring oral health related injuries, sickness and absenteeism; working with school health services to assess oral health status; advocacy and lobbying for oral health prevention; community engagement in screening; parental education and as role models [3, 8]. However, schools and teachers have limited capacity in dealing with oral health issues. A WHO global survey of 108 evaluations of school oral health projects across 61 countries reported that financial barriers (44%), limited capacity and availability of human resources (38%), lack of collaboration at lower levels (30%) and inadequate policy framework (24%) are the main barriers to school-based oral health activities [9].

Oral diseases can be prevented by addressing modifiable risk factors using low-cost interventions, thereby improving the health of children. Poor oral hygiene behaviour and unhealthy diets are known risk factors for developing oral diseases [3, 10]. Providing children with adequate knowledge and skills, including supervised tooth brushing, is necessary to promote children’s oral health and prevent oral disease.

A small number of studies has been carried out on teachers’ contributions to oral health promotion in national contexts of LMICs, with one report from India [6]. Also, there has been very little data analysing the implementation of the WHO oral health school framework in LMICs. A quantitative study in India reported that the majority (83%) of school teachers gave education about oral health topics [6]. School teachers were assessed on whether the curriculum has oral health topics, having been trained to deliver oral health topics, providing oral health education and the methods used. But school teacher’s contributions to oral health policy development, monitoring oral health conditions, and advocacy for oral health were not assessed.

Limited research has been done in Uganda. A study in Uganda established four health promoting schools in rural communities based on a successful Canadian health promotion initiative designed to address poor oral health in Canadian Aboriginal children in rural and remote communities [11]. After 4 years of inclusion of oral health topics in regular classroom activities, reinforcement of key educational concepts and daily in-school tooth brushing to develop healthy practices, Macnab and Kasangaki, (2012) [11] describe teachers’ perceptions on the impact of a health promoting school program in this rural setting. Analysis of teacher interviews showed there was support for the program, community engagement, benefits from the program, changes in policy in the school and challenges with implementation. In contrast to previous work, this study did not explore the mechanisms through which teachers engaged communities, participated in policy development and delivered oral health education. Another study in Uganda reported that only 42% and 52% of 12-year-old children in urban and rural areas of Gulu District, respectively, practiced the recommended twice daily tooth brushing [12]. No information was provided about their participation in school based oral health education.

This study aimed to explore the contribution of primary school teachers to oral health promotion in urban and rural areas in the Gulu District, Northern Uganda. No studies have focused on teachers’ contributions to oral health promotion. With current reports from Uganda indicating that only 1 in 2 children brush their teeth twice daily [12], it is important to understand current situation to improve the status quo. The views of school teachers on their contributions to oral health promotion are important to identify actions for schools and the community to promote oral health among children. Therefore, the research question being addressed was: what contributions have primary school teachers made in oral health promotion in rural and urban schools in Gulu district, northern Uganda?

This paper explores teacher contributions to oral health promoting in an existing school program following the WHO health-promoting school framework. This framework provides several responses to oral health needs of children and features six key components presented and described in Table 1 [8, 19].

**Table 1:** The six key components of the WHO’s health-promoting school framework and their description [8, 19]

WHO’s health-promoting school framework	Description of component
Engage health, education and community leaders	Engages health and education officials, teachers, teachers’ unions, students, parents, and community leaders in efforts to promote health
Improve health promoting policy and practice	Policy and practices that help create a healthy psychosocial environment for students and staff such as policies on healthy eating, adequate water and sanitation, first aid, curriculum to include oral health teacher training in oral health and prevention of oral diseases, assessment of oral health status, screening and treatment of oral disease and parental involvement in oral health programs
Provide a safe healthy environment	Provide supportive school environment, both physical and psychosocial including social support and mental health promotion, safe water and sanitation, teachers as role models, peer reinforcement and opportunities for physical education and recreation
Provide skills-based health education	Curricula that provide oral health education to help children develop personal lifelong skills, raise health consciousness, improve understanding and healthy attitudes, to promote healthy behaviours, and thereby to reduce risks of oral disease. Includes training and education for teachers and parents
Provide access to health services	Services like screening, dental examination, treatment and monitoring, and referral that may be provided in the school setting or in partnerships with other health agencies
Improve health of the community	Focus on community health concerns and participating in community health projects

Findings from this study are compared to current policy, practice and theory, thereby identifying gaps that need to be filled to improve oral health promotion activities in schools.

### Study design

We conducted qualitative descriptive study using semi-structured interviews with primary school teachers in rural and urban areas of Gulu District, Northern Uganda.

## Methods

Gulu is recovering from the impact of nearly 2 decades of civil war. The District Education Office (DEO) Gulu, provided a letter of support for the study and a list of all schools with contacts of headteachers. These headteachers provided names and telephone contacts of eligible participants who were contacted, and appointments made for interviews which aided gaining trust and rapport.

Informed consent was obtained from all participants. We provided participants with information on the purpose of the study and they were given opportunity to ask questions and seek further information before being asked to consent to participate in the study. Interviews with 18 participants were conducted by PA between December 2020 to April 2021. One participant declined to take part in the study because of other engagements. PA is a trained dentist and lecturer in public health at the Faculty of Medicine, Gulu University. He has extensive experience in designing, conducting, documenting and analysing interviews as a method and understanding how people interpret health and the contexts in which they occur. He is supported by a supervisory team with extensive qualitative research experience.

Semi structured interviews that lasted for 20–47 min (average 30 min) were carried out to gain a deep understanding of teachers’ views on oral health and their contributions to oral health promotion [13], as they have first-hand knowledge of oral health promotion in schools. We purposefully chose teachers with variation in age, gender, rural or urban location of school, level of education, subjects taught and years in service to gain a broad understanding of their contributions to oral health promotion.

Semi-structured interview guides were developed from WHO guidelines on oral health promotion through schools [8]. Prompts were used to explore areas of interest to the participants in greater detail. Probes and modifications to our interview guide where used throughout the process to ask more what one teacher said from the other teachers and questions were amended as new themes emerged. Semi-structured interviews gave participants freedom to share experiences. The interview guide was piloted with three primary schoolteachers to test participant acceptability and gain feedback on the questions. Memos and analytical notes were taken to track reflections of the research and record emerging themes, patterns and interpretations. All interviews were conducted face-to-face during the Covid19 pandemic in line with the guidelines for prevention of COVID-19 in a private location such as classrooms, school compounds and offices, without compromising the rights, welfare and safety of both participants and the researcher and they were digitally audio recorded after obtaining informed consent. The use of online platforms was considered; however, this was found inappropriate because of limited access to such technology by potential participants.

Participants’ responses were transcribed verbatim and checked against the audio recording for accuracy by a person independent of the research team; all transcripts were anonymised. Data collection and analysis occurred concurrently to generate understanding of the research question. Data collection continued until a point where information was being repeated by different participants. The decision to stop data collection was reached through discussion between authors.

### Data analysis

Thematic analysis was conducted as described by Terry et al., 2017 [14]. This involved familiarisation and coding of data, theme development, reviewing and defining themes, and report writing. Transcripts were read repeatedly to gain an insight into the data and generate codes. Candidate themes were developed and refined from codes with similar key features. A thematic map was developed and used to identify and understand potential themes. Research team members defined and named candidate themes after shaping, clarifying or even rejecting themes to ensure the themes worked well in relation to the coded data, the dataset and the research question. This involved writing an analytic narrative that encased the presented data extracts, providing a theme definition; short summaries of the core idea and meaning of each theme. Data were shared with some of the participants to check meaning and interpretation. Key themes and the analytic framework were shared and discussed at supervision meetings with all authors.

## Results

### Characteristics of study participants

Interviews were completed with 18 participants. Age of participants ranged from 30 to 52 years (mean age 40 years). Most of the participants taught more than one subject; mathematics and integrated science was the commonest subject combination. Participants had taught primary school children on an average of 14 years. Interviews lasted for 20 to 47 min (average 30 min). Characteristics of participants are presented in Table 2.

**Table 2:** Participant characteristics

ID	Age	Gender	Name of School	Subcounty)	Location	Years in service	Subjects teaching	Level of education
01	48	Male	Gulu Baptist Primary School	Layibi Division	Urban	24	English	Diploma
02	47	Male	Gulu Baptist Primary School	Layibi Division	Urban	18	Mathematics, Social studies and Science	Certificate
03	40	Female	Gulu Prison Primary School	Layibi Division	Urban	18	Integrated science and English	Diploma
04	36	Male	Credo Primary School	Laroo Division	Urban	15	Mathematics and Integrated science	Diploma
05	41	Male	Pece Prison Primary School	Laroo Division	Urban	15	Mathematics and Integrated science	Diploma
06	45	Female	Gulu Public School	Layibi Division	Urban	18	English and Social studies	Bachelor’s degree
07	32	Male	EL Shaadai Primary School	Bardege Division	Urban	9	Integrated science	Diploma
08	39	Male	Gulu Primary School	Bardege Division	Urban	16	Mathematics and integrated science	Diploma
09	52	Male	Obiya West Primary School	Bardege Division	Urban	18	Mathematics and Integrated science	Diploma
10	32	Male	Akonyibedo Primary School	Unyama Subcounty	Rural	10	Mathematics and Integrated science	Diploma
11	43	Female	Akonyibedo Primary School	Unyama Subcounty	Rural	15	All subjects	Advanced level
12	45	Male	Gwendiya Primary School	Awach Subcounty	Rural	15	Social studies	Diploma
13	34	Female	Panykworo Primary School	Bungatira Subcounty	Rural	10	Mathematics and English	Diploma
14	30	Male	Palaro Primary School	Palaro Subcounty	Rural	4	Mathematics, Integrated science, Religious education	Diploma
15	40	Female	Angaya Primary School	Unyama Subcounty	Rural	14	Mathematics and Physical education	Bachelor’s degree
16	47	Male	Cwero Primary School	Cwero Subcounty	Rural	23	Mathematics and Integrated science	Bachelor’s degree
17	37	Female	Omoti Ills Primary School	Patiko Subcounty	Rural	14	Social studies	Diploma
18	40	Male	Kulu Keno Primary School	Bungatira Subcounty	Rural	9	Integrated science	Diploma

Seven core themes were developed from the data against the WHO health promoting school framework. The first theme, provide skills-based health education, comprised two subthemes. The theme described contributions teachers have made to help children develop skills and raise health awareness to prevent oral disease. The second theme, provide access to health services, comprised of three subthemes. This theme highlighted contributions teachers made in dental examinations, treatment, monitoring and referral. The third theme, engage health, education and community leaders, comprised three subthemes. This theme analysed contributions teachers made towards engaging parents, students and health workers. The fourth theme, providing a safe healthy environment, comprised three subthemes. This theme analysed contributions teachers made towards providing social and psychological support, water and sanitation and inspection of school requirements. The fifth theme, improve health promoting policy and practice, included three subthemes. This theme describes contributions teachers made towards supervised tooth brushing, inspection of school requirements, and rewarding good performance. The sixth theme, improving health of communities, included two subthemes. This theme describes contributions teachers made towards community health concerns and participation in community health projects. The last theme, response to the corona virus disease of 2019 (COVID-19) pandemic, describes teachers’ contributions to oral health promotion in schools that were altered and those that were not interrupted. The following sections describe these themes and subthemes with their quotes. The themes and subthemes are presented in Table 3.

**Table 3:** overview of themes and subthemes with illustrative quote

Theme	Subtheme	Illustrative quotes
Provide skills-based health education	Developing skills	“….but as a teacher before even in class you stand, you get a stick, you tell them this is how we brush. You take upside down the other part the inner part and so on”. Interview 10
		“……there is also a topic in p2 which when you are teaching you should demonstrate the style of brushing teeth”. Interview 16
		“The teacher will get a brush, will ask learners to go and get some small-small sticks to come and make your own brush then the teacher will demonstrate how to brush properly. So that the learners know how to brush”. Interview 17
	Raise health awareness to prevent oral disease	“they talk about types of the teeth that is; milk teeth, permanent teeth all those the molars the premolars, incisors, diseases of teeth, how to control them they are there in that syllabus”. Interview 03
		“I think from school here we try our best to advocate for the hygiene of the mouth, I could say it, we encourage them to brush their teeth regularly, after meals”. Interview 12
		“the tooth decay I may tell them that you can eat things like biscuits, sweets, sugar cane but not in large amount. Just eat so that I helps your body to…to have nutrients but know that if you eat too much of it, you are likely to get tooth decay because that germ likes things which are sweet and if you don’t brush your teeth the food remains are going to remain in your…….if you don’t keep on brushing they are piling and now the germs will get a way of entering and then you will automatically get tooth decay”. Interview 17
		So that one, you know normally we follow curriculum. So that curriculum about teeth falls under science so strictly it is done under science but now you will bring others in the class when you, it will not even stop you, like when you want to talk about how they should keep their teeth clean and so on. You can bring that like interlude in your lesson. You know for us teachers you just bring them as if it is an interlude. Interview 03
Provide access to health services	Dental examination	Yeah. We have what is called morning health parade. In the morning, if the schools are running normally, in the morning parade that is where you have to check on the hygiene of the child: the mouth, the fingernails, the hair if it is clean, even the clothing but we only advise. Interview 11
	Treatment, monitoring and referral	“But now for us what we could do was just to refer them because we don’t know anything which is causing those kinds of things. So, we could…. if it is about the bad smell yes, we could fight it but what is happening…. what is causing that thing to….to bleed we do not know, so we need to refer them. We just say ahh please the parents, this thing is like this can you take the child to medical personnel so that is what we are doing”. Interview 05
		“….., like when a child has not brushed, you can even see and identify it. Then you send that child to brush immediately, you send them, you just get…. You just say, let us go and get some sticks, these sticks, you break and then you use it, and at least you see at least it is fairly done”. Interview 1
Engage health, education and community leaders	Engaging parents	“Yeah, when we organise parent meetings, we give that sensitisation to them and during athletics when we gather a large number of parents, so we pass the message…….”. Interview 16
		“.it is one of our priority here that we must make sure that if any problem is there that we have already noticed….we need to share with the parents…. others could go up to the SMC and the PTA. In their meeting it is also presented, the school is having this and that, what should we do?” Interview 05
	Engaging oral health professionals	“Like sometimes we have been hosting those ones from the health centre there, they can come here. They train these pupils. Like all teachers are called to sit together with the children then they help those health workers to explain certain things to the children” Interview 14
		“ We invited a dentist…, who came here and checked most of our children especially their teeth, the gums and the rest of it…”. Interview 02
	Engaging other school staff	“……., you pick one child then you get clean water you go may be under the tree then the child, you demonstrate to the child what he does from home”. Interview 02
		“Yes, we have noticed that it started reducing because we found it, it was already a problem then we had to tell them exactly what they are supposed to do like: they are supposed to brush regularly, and also……”, Interview 09
Provide a safe healthy environment	Provide social and psychological support	The health club is a club which is formed by these students and they have a matron and patron and then they have to look for activities around to keep the health of the learners, that is, we have a team with the chairperson and then the other members in that structure whereby we also draw a program for them weekly. Interview 15
		I feel it…. and it is very important because if…….I knew if I visited……if my father had taken me to a dentist other than extracting the tooth they would have refilled, and I would not have lost the tooth. But I lost the tooth then when I become older myself when I visited a dentist, I got better knowledge, so I choose to now follow the dentist advise other than removing a tooth…………………, and I always refer to myself. Had I not been following I would have lost many teeth. Interview 04
	Provide water and sanitation	“We have got water, sanitizers, we check on their latrines. We check everywhere. Of course, even as we shall get out you get, we see we have talking compound as they are in the compound, they continue learning” Interview 01
		“Like we have water even: we have the borehole and there are washing facilities also, uhm like for washing their hands. Yeah, the area for brushing is also there”. Interview 06
	Act as role models	“I feel it…. and it is very important because if…….I knew if I visited……if my father had taken me to a dentist other than extracting the tooth they would have refilled, and I would not have lost the tooth. But I lost the tooth then when I become older myself when I visited a dentist, I got better knowledge, so I choose to now follow the dentist advise other than removing a tooth…………………, and I always refer to myself. Had I not been following I would have lost many teeth” Interview 04
Improve health promoting policy and practice	Supervised tooth brushing	“now when it comes to another strategy that we are doing especially last term……., we make sure that in the morning you must check them to make sure that everybody brushes [their teeth]”, Interview 05
		“We try to follow up on them like the matrons should monitor them to make sure that each and every one does the brushing in the morning before going to class like that”. Interview 07
	Inspection of school requirements	“The restrictions like we, those sweets in most cases we don’t encourage parents to pack for their children because the things that we normally encourage them to pack for their children are things like, some maybe appetizers: you can put “odii” for them that one there is no problem. So mainly those are the things we look at but issues to do with sweets what, what, we discourage parents to pack for their children”. Interview 07
		“Now as the school the school tried to put some restriction that learners must not come with extra sugar in their cases. Sugar is always collected. The parents will bring sugar. Like when you get at the gate, you find that the teachers…… you have seen”. Interview 08
	Rewarding good performance	“One example, you can cite that child as……you give that child as an example among the pupils. You say, you see a good……a clean child looks like this….like this one. You can select the best girl, the best boy, then you can motivate them, you talk to them, you make the……., children will start learning and change”. Interview 14
		“Yeah, rewards in terms of appreciation like we speak positive things about that child…….” Interview 12
Improving health of communities	Focusing on community health concerns	“…….most of our children here suffer from this tooth ache because of eating sweet things, they like carrying sweets, eating biscuits. So you find them staying for…. after eating they don’t even brush, even from school here you need to remind them even to do [brush] ………” Interview 02
		“You are a teacher, you are marking, and a child comes to you, a child is explaining something to you, automatically you will know that this child did not brush” Interview 03
		“Yeah. A number of times we have been recording learners suffering especially from tooth ache, the most common one is tooth ache. And sometimes bad breath”. Interview 08
	Participating in community health projects	“We invited a dentist……., who came here and checked most of our children especially their teeth, the gums……… we were also advised by him that we could constantly keep on checking those children in case we get anything we have to report to them so that they come here” Interview 02
		“Like sometimes we have been hosting those ones from the health centre there, they can come here. They train these pupils……… sometimes children don’t see what you are telling as a teacher that it is true, so they first want to see from the medical workers. When they come here, they can handle and supplement on what the teacher has been doing.” Interview 14
Response to COVID-19 pandemic	What has changed	“……maybe because of this COVID-19 but previously we used to have these sessions.” Interview 07
		“Hmm. since we started this year, we have not involved them [parents]” Interview 15
		“Yeah. One, we have, like we have before “corona” we were having what we call health club. Yeah. We always giving assembly we like to…like this oral health. Yes. We try to teach them how we can wash our hands, how we can also brush our teeth. We always do because some people they …… some of them they brush their teeth just for one minute it is over. Yes, but we were just teaching them before corona but after corona we have not yet done”. Interview 18
	What was not interrupted	“We told them they should brush at least after every three….after every meal, at least three times in a day because very early before coming to school they brush their teeth, then from here like we have a boarding section also here, and the candidates were all in boarding because of this COVID so we would also encourage them to brush in the morning” Interview 06
		“Yes, we tell them, this one normally comes in when we are teaching, when we are handling science and it comes to that part of food and nutrition, we tell them and when it comes to human health,……teaching on the parts of the teeth, the human teeth; the causes of tooth decay; how to avoid tooth decay; care for the teeth. We all bring in those ones”. Interview 10
		“….., we ask the parents to provide their children, especially those we cater for as boarders, to provide them enough things they use for cleaning themselves especially the mouth cleaning. So, we ask them to provide us enough toothbrushes…. the required toothpaste also. Interview 04

### Theme 1: provide skills-based health education

#### Developing skills

Teachers prepared children to practise proper oral hygiene care through skills training. Many teachers performed demonstrations on how to brush teeth and discussed the materials used for brushing.

The teacher will get a brush, will ask learners to go and get some small-small sticks to come and make your own brush then the teacher will demonstrate how to brush properly. So that the learners know how to brush. (Interview 17)

#### Raise health awareness to prevent oral disease

Teachers mentioned their role in raising health awareness by providing information on causes and prevention of oral diseases. A few teachers provided information on the consequences of oral diseases such as cavities and pain in order to discourage children from habits that cause oral diseases.

I tell them that you can eat things like biscuits, sweets, sugar cane but not in large amount. Just eat so that it helps your body to…to have nutrients but know that if you eat too much of it, you are likely to get tooth decay because that germ likes things which are sweet and if you don’t brush your teeth the food remains are going to remain in your…….if you don’t keep on brushing they are piling and now the germs will get a way of entering and then you will automatically get tooth decay. (Interview 17)

In contrast to providing information on consequences of oral disease, one teacher mentioned providing children with information on the benefits of maintaining oral health. He told his students good oral health will help build their confidence.

Teachers mentioned providing oral health education as part of the curriculum, mainly through science, but also integrated into other subjects. Teachers delivered the curriculum using different pedagogical styles. A few teachers mentioned asking a series of questions, to elicit critical thinking, reasoning and logic, as a method of delivering topics in the curriculum. Many teachers mentioned children were taught in classes, assemblies and health parades where they are made to make connections between structure and function of teeth, and the causes and prevention of oral disease, so that the information and skills can be applied to adopt healthy lifestyles.

Especially how to keep their teeth clean, things which can cause problems like eating sweet things, not brushing the teeth and ……., after every meal you are supposed to brush your teeth. (Interview 02)

### Theme 2: provide access to health services

#### Dental examination

Teachers mentioned they were involved in examination, treatment and monitoring of children’s health. As part of their duty many teachers performed routine health inspection at what they called “health parades”, once or twice a week, at assembly or during physical education lessons.

We have what is called morning health parade. In the morning, if the schools are running normally, in the morning parade that is where you have to check on the hygiene of the child: the mouth, the fingernails, the hair if it is clean, even the clothing but we only advise. (Interview 11)

At these health parades teachers make a visual inspection of children including their mouth. Teachers were able to identify gum disease, poor oral hygiene practices and tooth decay. However, teachers mentioned that a potential disadvantage of this open method is that some children are hesitant to have the examination because they are fearful of exposing their oral disease and hygiene status to teachers as it may result in seeking management of oral diseases or being asked to brush.

Through health parades, plans for further management of oral diseases and conditions have been informed including inviting dentists to school, informing parents and school administration of children’s oral health status and delivering guidance to children on proper oral hygiene. In one school, a dentist was invited to the school:

We invited a dentist..., who came here and checked most of our children especially their teeth, the gums and the rest of it…………. And after his coming we were also advised by him that we could constantly keep on checking those children in case we get anything we have to report to them so that they come here. (Interview 02)

Activities conducted during the health parade are linked to topics taught in the curriculum such as how to keep the mouth clean and primary health care. However, one teacher felt this was not enough and suggests a need to improve knowledge and skills of teachers in conducting health inspection.

I think, one, as teachers they were not well informed about problems related to oral health……... So, there is that lack of knowledge and yet it is a big problem, (Interview 01)

#### Treatment, monitoring and referral

When child health problems were identified, teachers make the decision to provide treatment at school or refer to a health facility. The most common treatment provided at school was simple pain relief or medication to reduce a high temperature.

For more complex cases, teachers talked about their role in referring children for better management at nearby health facilities. Referral was made by the teacher on duty or the teacher in-charge of health and sanitation by writing details of the child on a piece of paper or booklet. Teachers felt they did not have knowledge, skills and the mandate to treat some of the cases such as tooth loss due to accidents, tooth ache and bad breath.

But now for us what we could do was just to refer them because we don’t know anything which is causing those kinds of things. (Interview 05)

### Theme 3: engage health, education and community leaders

#### Engaging parents

Many teachers mentioned that they engaged parents to make the school a healthy place. Teachers involve parents in oral health promotion activities like providing tooth brushes and tooth paste, referral of children and oral health education. Parents received information on school requirements for oral hygiene, the burden and prevention of oral disease and were consulted on how to prevent oral disease. Parent days, school days, athletics days and meetings provided communication channels between teachers and parents.

Yeah it is one of our initiative here, we make sure that we involve parents fully. (Interview 05)

#### Engaging oral health professionals

Teachers mentioned engaging oral health professionals from government and non-government organisation in dental examination and referral of children, providing oral health education to children and teachers, providing oral hygiene materials to children. Engagement with oral health professionals has mainly been reactive, teachers often follow plans and activities that have been generated by these organisations.

Yeah, sometimes we invite people…….and once in a while we also invite health workers to give more. I mean the details of what we always emphasise. As teachers we may not go more into the detail of the personal or tooth decay…….., but in 2019 we had a team of health workers who came here and educated the learners so much on tooth decay and to some extent they also had somebody who gave them……toothpaste.

#### Engaging school children

Many teachers mentioned engaging in efforts to promote oral health of students in several ways including providing oral health education, examination, treatment, management, social support, access to safe water and participated in oral health projects.

In our school there is our school nurse for emergency, a teacher chosen by us [teachers]. ….that teacher sometimes gives them medicine…., the pain killer to reduce the pain…… (Interview 11)

### Theme 4: provide a safe healthy environment

#### Provide social and psychological support

Many teachers felt schools were the best place to deliver health promotion to children including providing social support, safe water and sanitation and teachers as role models. Teachers also mentioned they provided opportunities for children to get social support regarding health, learning and welfare.

The health club is a club which is formed by these students and they have a matron and patron and then they have to look for activities around to keep the health of the learners, that is, we have a team with the chairperson and then the other members in that structure whereby we also draw a program for them weekly. (Interview 15)

#### Act as role models

Teachers felt they could act as role models by setting a good example and encourage children to adopt a healthy lifestyle.

………if my father had taken me to a dentist other than extracting the tooth they would have refilled, and I would not have lost the tooth. But I lost the tooth ……., and I always refer to myself. Had I not been following I would have lost many teeth, (Interview 04)

#### Provide water and sanitation

Teachers mentioned they ensure children have access to water and sanitary facilities. Often doing inspection to check whether water is available, and the sanitary facilities are fit for use. Children were asked to bring items for their health and nutrition such as toothpaste, water bottles and sugar to school.

We have got water, sanitizers, we check on their latrines. We check everywhere. (Interview 01)

### Theme 5: improve health promoting policy and practice

#### Supervised tooth brushing

Teachers implement supervised tooth brushing, inspection of school requirements and rewarding good performance. Often checking that children have brushed their teeth in the morning, after meals and before going to bed.

Now when it comes to another strategy that we are doing especially last term……., we make sure that in the morning you must check them to make sure that everybody brushes [their teeth], (Interview 05)

#### Inspection of school requirements

Schools require that children bring items for their health and nutrition such as toothpaste, water bottles and sugar. Teachers mentioned they ensure that these requirements are fulfilled.

….we look at their [school] requirements: it is also one of their requirements that they must come with a toothpaste… (Interview 05)

#### Rewarding good performance

Teachers mentioned rewarding children for performing well during health assessments in form of words of encouragement to acknowledge good efforts as well as personal achievements.

Yeah, rewards in terms of appreciation like we speak positive things about that child but giving incentives like may be books, that one we haven’t done. (Interview 08).

### Theme 6: improve health of communities

#### Focusing on community health concerns

Many teachers mentioned children have oral diseases such as tooth decay, bad breath and gum disease that affects school attendance, socialisation, class performance and is a source of pain and discomfort. The other oral health issues of concern among children were consumption of foods high in sugar, not brushing, lack of parental guidance on oral hygiene and not visiting a dentist.

,what always I see affecting them especially this problem of the tooth decay, yeah. And at times they also have bad smell which makes at times learning difficult even to the teaches and their fellow pupils…. (Interview 05)

#### Participating in community health projects

Teachers mentioned they participated in oral health promotion projects consisting of health education, distribution of materials for oral hygiene, screening for and management of oral diseases.

They even gave us some chats how to educate these children. It was real education……... So after training us, we were moving from class to class. We give out, before giving out those things [tooth paste and tooth brush], we first give them that education we received……., then we distribute those things to them. (Interview 03)

### Theme 7: response to COVID-19 pandemic

#### What has changed

Many teachers mentioned that COVID-19 had changed the way they contributed to oral health promotion activities in schools. These included enforcing measures that limit interaction of children with fellow children, teachers and community. They reduced the number of oral health education sessions, parent and school days, engaging health workers in oral health promotion activities, health club activities and health assessments and restricted vendors from accessing school premises to prevent the spread of COVID-19.

Eh you know with this “corona” ah…there are SOPs; the guidelines that we were given. Like at the moment there are some other subjects that we are not supposed to teach. They are there, music is one of them, PE is also one of them, art and technology we are not supposed to teach. Any teaching that will bring children near (in close contact). (Interview 18).

#### What was not interrupted

Despite the challenges with promoting oral health by teachers during the COVID 19 pandemic there were some contributions teachers made. Many teachers mentioned they provided oral health education, conducted health parades, inspected for school requirements such a toothpaste and toothbrushes, referred of children for better management of oral diseases, supervised tooth brushing, provided social support, water and sanitation facilities, and involved parents involvement in addressing oral health challenges among children through the school management committee (SMC) and parent-teacher association (PTA).

we make sure…… that in the morning you must check them to make sure that everybody brushes [their teeth]. Two we look at their requirements: it is also one of their requirements that they must come with a toothpaste and they must also use it because of this COVID they said that hygiene should be maximum, so we are doing our best (Interview 05)

## Discussion

In this study documenting primary school teachers’ contributions to oral health promotion in Gulu, Uganda, we found that teachers were able to provide skills-based education, improve health of communities and promote healthy school environments. Based on the WHO’s health-promoting school framework we noted the key features of the framework were addressed, including providing skills-based education, safe health environment, access to health services, engaging health, education and community leaders and improving health of communities. Studies in other LMICs have documented similar findings, where teachers engaged in providing skills-based education [6, 15, 16], providing access to health services [17, 18], and engaging health, education and community leaders [18].

The WHO’s health-promoting school framework provides several responses to oral health needs of children and features six key components (Table 1) [8, 19]. Previous research has described various interventions based on this framework including oral health education sessions for children and mothers, integration of oral health education into curriculum, use of educational materials, active involvement of children, teacher training with follow up workshops for reinforcement, contests on oral health, tour of the dental hospital, oral examination and informing teachers and parents of the children’s dental health status, free fluoride toothpaste and treatment, daily supervised tooth brushing [20–22]. The majority of these interventions require active involvement of teachers, yet there has been little work done to understand the contributions of primary school teachers regarding oral health and the WHO framework.

In our study themes are derived against the WHO health promoting school framework. The first theme, “provide skills-based health education” is congruent with regional and national strategies for improving oral health and the primary school curriculum in Uganda [3, 5, 23]. According to several theories, increasing awareness is the key first step necessary for behaviour change [24]. Similar findings on providing oral health education have been documented among teachers in other LMICs such as Nigeria, China, and India [6, 15, 16]. While previous research has focused on the proportion of teachers who provided oral health education to children, our findings demonstrate that teachers contributed to skills training and raising health awareness.

Teachers were involved in improving oral health of communities by performing examination, treatment, referral, epidemic response and work with communities with some challenges noted. Similar findings regarding provision of health services are reported in other LMICs. A questionnaire-based study in Nigeria reported several primary school teachers were engaged in tooth cleaning and inspection of children [17], whereas our findings demonstrate that teachers are also involved in dental examination, treatment, referral and responded to the COVID-19 pandemic. Additionally, we identified that reduced oral health-care and promotion in schools has been a previously unforeseen outcome of the COVID-19 pandemic.

Teachers engaged communities in oral health promotion. This is similar to findings from a descriptive cross-sectional study among 511 teachers in India [21], which showed 55% of teachers discussed oral health of children with their parents during meetings. Working with communities is vital for ownership, support and sustainability of public health interventions. However, we found little evidence of partnership with the community on each aspect of oral health promotion from development (community involvement) and we could not identify instances of final decision making on oral health promotion at the community level (shared leadership) and engagement with community leaders.

In keeping with “provide a safe healthy environment”, of the WHO’s health-promoting school framework, our study provided new insights into teachers’ roles in promoting healthy school environment through providing social and psychological support, safe water and sanitation and inspection of school requirements. Perhaps promoting healthy school environment for children may have some benefits on oral health although not many teachers mentioned being involved in promoting healthy school environment.

Oral diseases remain a public health challenge in Uganda, affecting 30–70% of school children and oral health practises among school students are deficient [12, 25]. Lack of awareness, limited access to oral health services, poor hygiene practices and consumption of unhealthy diets high in sugar have been identified as major contributors to the high prevalence of dental caries in Uganda [5, 12]. Our study found that teachers’ involvement in oral health education and dental assessment of children is in accordance with the national oral health policy of Uganda. Teachers provide children with knowledge and skills, access to oral health services and a healthy school environment necessary to promote children’s oral health and prevent oral disease. However, many teachers expressed a lack of knowledge and skills in oral health education and dental assessment of children. Teachers’ contributions are an important issue when considering promoting oral health using the health promoting school’s framework. Studies that have used teachers in delivery of oral health interventions have reported reduction in dental caries, plaque and improvement in oral health knowledge attitude and practices among school children [11, 20, 21, 26, 27]. One of the proposed strategies for improving oral health in Uganda is implementing a school oral health program that includes oral health education, periodic screening of children and training teachers on oral health education and promotion harmony with the school health program [5]. Our study has identified contributions teachers have made to oral health promotion in schools, however there are potential challenges and barriers to such a program, and further research is needed to identify these challenges and barriers and the most appropriate resources and training needs for teachers promote oral health in schools.

The implications of this study are that teachers’ contributions to oral health promotion were aligned to the WHO’s health-promoting school framework. Several contributions made by teachers and opportunities for interventions were noted including skills training and raising awareness, performing examination, treatment, referral, epidemic response and engaging parents and health workers.

It is feasible to use teachers in interventions to promote oral health among primary school children, however regular training could be needed to improve skills-based education and providing oral health services. In addition, engagement of parents, health workers, and community leaders is necessary for design and implementation of interventions promoting oral health among school children. This will require local, district and national support and resources for oral health promotion for the benefit school children.

### The strengths and limitations of this study

The background of the first author is in dental surgery and public health (PA). He has been involved in oral health promotion and management of oral conditions in the region for the past 20 years. His experience and professional beliefs could have impacted on the questions asked during the interview and how he viewed the data collected. However, as per best practice, he kept a log of his thoughts and feelings during and after the interviews. In addition, themes were discussed with the wider study team that were non-dental in training.

Teachers were trained educationists with several years of delivering the primary school curriculum. This could have influenced the way they answered questions. Their responses to questions could have been biased towards the ideal or right answers rather than what was happening on the ground. However, by purposefully selecting a diverse study population and reporting on prominent and unusual themes the study delivers a deep understanding on the views of teachers sampled. In addition, purposeful sampling of teachers in Gulu District limits generalisability of our findings to other settings in Uganda. Further limitations are that headteachers may have had bias in providing the list of candidate interviewees and participants were not asked to provide feedback on the study findings.

## Conclusions

Teachers can be a valuable and skilled resource in oral health promotion in resource poor settings. Teachers are implementing key principles of the WHO’s health-promoting school framework on the ground and need to be considered as a key public health resource. If improvements in oral health are to be attained in Sub-Saharan Africa and other LMICs, government interventions need to harness teachers’ contributions in delivering oral health promotion. The WHO’s health-promoting school framework is a useful way of assessing teachers’ contributions to oral health promotion in schools.

## Data Availability

The data that support the findings of this study are available from University of New South Wales (UNSW), but restrictions apply to the availability of these data, which were used under license for the current study, and so are not publicly available. Data are however available from the corresponding author upon reasonable request and with permission of UNSW.

## Abbreviations

COVID-19: Corona virus disease of 2019
DEO: District Education Office
LMICs: Low-and middle-income countries
NCD: Non communicable disease
PTA: Parent–Teacher Association
SMC: School Management Committee
WHO: World Health Organisation
